# An unlabelled probe-based real time PCR and modified semi-nested PCR as molecular tools for analysis of chloroquine resistant *Plasmodium vivax* isolates from Afghanistan

**DOI:** 10.1186/s12936-020-03323-4

**Published:** 2020-07-14

**Authors:** Sayed Hussain Mosawi, Abdolhossein Dalimi, Najibullah Safi, Reza Fotouhi-Ardakani, Fatemeh Ghaffarifar, Javid Sadraei

**Affiliations:** 1 Department of Medical Parasitology, Faculty of Medical Sciences, Tarbiat Modares University, Tehran, Iran; 2 Medical Sciences Research Center, Ghalib University, Kabul, Afghanistan; 3 World Health Organization Country Office, Kabul, Afghanistan; 4 Cellular and Molecular Research Center, Qom University of Medical Sciences, Qom, Iran

**Keywords:** *Plasmodium vivax*, *pvmdr*-*1*, *pvcrt*-*o*, Chloroquine resistance, Afghanistan

## Abstract

**Background:**

*Plasmodium vivax* resistance to chloroquine (CQ) has been reported from many endemic regions in the world. *Plasmodium vivax* is responsible for 95% of malaria cases in Afghanistan and CQ is the first-line treatment given for vivax malaria. The *pvmdr*-*1* and *pvcrt*-*o* (K10 insertion) genes are possible markers for CQ-resistance in *P. vivax* isolates. There have been no studies done on the presence or absence of molecular markers for CQ-resistance *P. vivax* in Afghanistan. The present work aimed to evaluate the frequency of mutations in the *pvmdr*-*1* and K10 insertion in the *pvcrt*-*o* genes of *P. vivax.*

**Methods:**

*Plasmodium vivax* isolates were collected from Laghman, Baghlan and Khost provinces. For investigation of polymorphisms of desired regions in *pvmdr*-*1 and pvcrt*-*o* genes, sequencing was applied on the PCR products. A new asymmetric qPCR and melting analysis assay based on unlabelled probe developed for scanning of K10 insertion in *pvcrt*-*o* gene.

**Results:**

The analysis of sequencing data of the *pvmdr*-*1* gene showed wild type Y976 and K997 and mutant M958 and L1076 in 33 isolates from three provinces. Of the 36 samples evaluated for K10 insertion in *pvcrt*-*o*, 2/18(11%), 0/10(0%) and 0/8(0%) isolates from Laghman, Baghlan and Khost province, respectively, possessed K10 insertion, confirmed by either sequencing and unlabelled probes.

**Conclusion:**

Two samples with K10 insertion and 33 samples with *pvmdr1* polymorphism, indicating on the possibility of CQ resistance in *P. vivax* populations in Afghanistan. Furthermore, unlabelled probes are simple and inexpensive alternative tools for screening of *P. vivax* mutations.

## Background

About 216 million cases of malaria were reported from 91 countries during 2016. Afghanistan has 13% (138,217) of malaria cases in the eastern Mediterranean region and 95.6% (132,237) of these cases were vivax malaria [1]. Because of its relatively low mortality, *Plasmodium vivax* is second in priority among the causative agents of malaria in the world, and has the widest geographical distribution when compared with the other species of human malaria parasites [2]. Most tropical areas, including the Middle East, Asia, and the Western Pacific, account for about 80–90% of vivax malaria outside of Africa [3]. Although severe malaria (characterized by cerebral malaria, renal failure, circulatory collapse, severe anaemia, haemoglobinuria, abnormal bleeding, ARDS, and jaundice) is mainly caused by *Plasmodium falciparum*, there is now accumulating evidence showing that *P. vivax* can also cause severe malaria in humans [4]. The first reports of chloroquine (CQ) resistant malaria was published in1959 for *P. falciparum* and in 1989 for *P. vivax*, when the malaria CQ-resistance studies were started in Afghanistan [5–7]. The *P. falciparum* CQ resistance was very worrying issue at the time, since it took many lives in several countries, mainly in Asia. Therefore, to avoid having the same situation with *P. vivax*, extensive, rapid and wide research must be performed [8]. Some of main causes for failure to control and eradicate vivax malaria are the emergence of anti-malarial drug resistant strains and their ability to become dormant stages that could later on relapses weeks or months after the initial infection [9].

The first cases of chloroquine-resistant *P. vivax* were reported in Papua New Guinea and after that many more cases were observed in other parts of the world [5]. While reports of CQ-resistant *P. vivax* infections are increasing, this drug is still the primary therapy for vivax malaria in many endemic countries [6]. Chloroquine has been widely used in the treatment of uncomplicated malaria in Afghanistan since the 1940s, and remains the most widely used anti-malarial drug [10].

Although there are few studies have been done to assess CQ resistance, no work has been done to evaluate CQ efficacy on vivax malaria in Afghanistan [11–13]. Many studies have been performed to analyse the single point mutations in the *pvmdr1* gene on chromosome 5 and *pvcrt*-*o* gene on chromosome 1, and to evaluate their relation to the resistance of *P. vivax* to chloroquine [14]. Some studies have failed to demonstrate an association between *pvmdr*-*1* and *pvcrt*-*o* mutations with the CQ resistance in *P. vivax,* and further investigations are needed to confirm these associations [15, 16]. Research studies in some parts of the Brazilian Amazon, analysed polymorphisms within the coding and noncoding sequences of *pvmdr*-*1* and *pvcrt*-*o* genes and the copy number variation in the *pvmdr*-*1* gene [14, 17]. Melo et al. showed that patients with CQR showed an increase up to 6.1-fold and 2.4-fold in *pvcrt*-*o* and *pvmdr*-*1* expression levels, compared to the susceptible population in this region that highlighted the association of *pvcrt*-*o* and *pvmdr*-*1* with CQR *P. vivax* malaria [18]. Other studies also supported the effect of *pvcrt*-*o* gene on CQ transportation or accumulation by *P. vivax* [19]. Suwanarusk et al. [20] also showed an association between the CQ susceptibility of *P. vivax*, the amplification of the *pvmdr*-*1* gene and the relevant SNPs. Brega et al. found polymorphisms at two different codons of the *pvmdr*-*1* gene (positions Y976F and F1076L) [21]. Another study in Madagascar, showed the possible relationships between the *pvcrt*-*o*, *pvmdr*-*1* genes and the clinical responses of the patients to CQ treatment [22]. Some studies reported that the Y976F mutation in the *pvmdr*-*1* gene were associated with a reduction in CQ susceptibility of *P. vivax* isolates in vitro [23, 24]. Some reports suggest the Y976F and the F1076L polymorphisms are common in Latin America [25]. Ganguly et al. investigated the prevalence of *pvcrt*-*o* and *pvmdr*-*1* gene polymorphisms and the in vivo efficacy of CQ in *P. vivax* isolates from India [26]. So far, such studies have not been done in Afghanistan. There are several methods to analyse point mutations such as PCR-sequencing, ARMS-PCR, PCR–RFLP. One of the new techniques for the detection of polymorphisms of genes is asymmetric qPCR and using unlabelled probes [27]. In this study, for the first time the insertion of K10 in the *pvcrt*-*o* gene was investigated using a modified semi-nested PCR-sequencing approach and an asymmetric qPCR method. Successful and accurate detection of drug-resistant *P. vivax* parasites may be helpful in malaria control programmes, as well as treatment and elimination strategies [28]. This work aimed to evaluate the mutations of CQ-resistant associated genes in *P. vivax* isolates from three endemic provinces of Afghanistan.

## Methods

### Locations, *Plasmodium vivax* samples collection and DNA purification

**Fig. 1:**
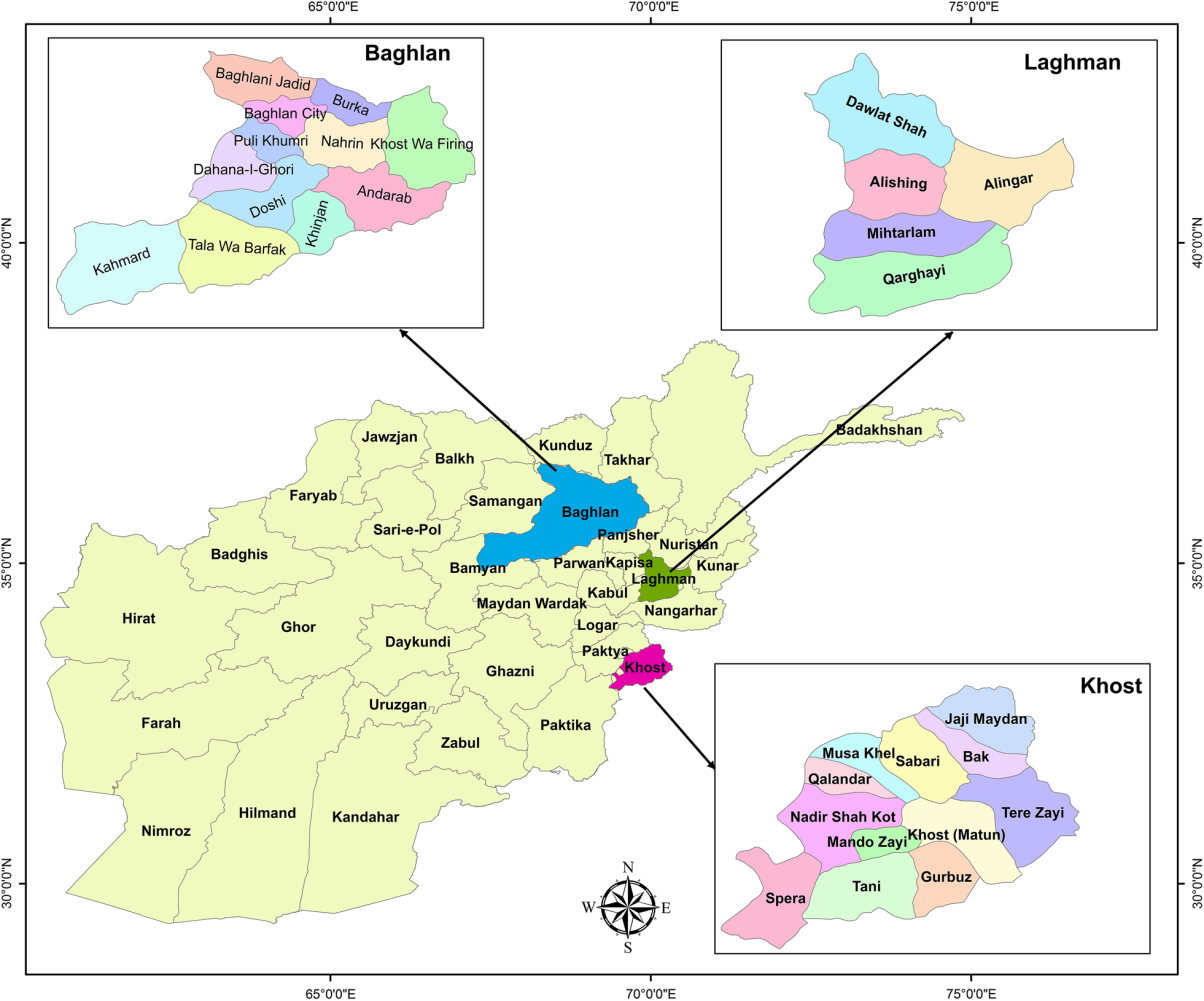
Geographical map of Laghman, Baghlan and Khost provinces in Afghanistan

In general, 50, 15, and 15 *P. vivax* microscopically confirmed cases were collected from the patients that were referred to malaria diagnostics centres in Laghman (34° North, 70° East), Baghlan (36° North and 68° East) and Khost (33° North and 70° East) provinces (Fig. 1), respectively, during 2017. For this, finger stick blood samples were spotted on DNA Banking Cards (DBCs) (Kowsar Biotechnology Center, Tehran, Iran) and microscope slides.

### DNA extraction and *Plasmodium vivax* confirmation

Disks (2 mm in diameter) were punched out from each DBCs and washed 3 times with DBCs purification buffer and twice with distilled water. The disks were air dried and used directly as template of PCR processing [29]. A semi-nested multiplex PCR were performed on each sample for final confirmation of *P. vivax* [30].

### Evaluation of *pvmdr1* mutations by nested-PCR

Briefly, in first round *pvmdr*-*1* (OF) and *pvmdr*-*1* (OR) primers (Table 1), were applied for amplification of about 967 bp fragment of *pvmdr*-*1* gene of *P. vivax*. Three punched discs were used for each PCR reaction of the first round. One microlitre of all products of the first round diluted into 500 μl of sterile water. In the second round 1 μl of *pvmdr*-*1* (NF) and *pvmdr*-*1* (NR) primers (Table 1) and 2 μl of diluted product of the first round were added into 25 μl of “2X Taq Master Mix Red” (Amplicon inc., containing 150 mMTris-Cl pH 8.5, 40 mM (nh4) 2So4, 3 mM Mgcl2, 0.2% tween 20, 0.4 mM dntPs, 0.05 U/µl Taq DNA polymerase, inert red dye and stabilizer), to reach a final concentration of 50 μl. The first round was done in 94 °C, 5 min; 30 cycles of 94 °C, 15 Sec; 60 °C, 30 Sec; 72 °C, 1 min; 72 °C, 7 min, while the second round PCR was done under the following conditions: 94 °C, 5 min; 30 cycles of 94 °C, 15 Sec; 57 °C, 30 Sec; 72 °C, 1 min; 72 °C, 7 min. The products of the second round (604 bp) were seen in 2% agarose gels (SYBR Safe stain; Invitrogen; Groningen, The Netherlands). The *pvmdr*-*1* (NF) and PCR products were sent for sequencing by the ABI3730XL sequence analyzer (Macrogen, Korea) [8].

**Table 1:** Primers used for amplifications of *pvcrt*-*o* and *pvmdr*-*1* marker genes: OF (Outer forward), OR (outer reverse), NF (nested forward), NR (nested reverse)

Primers	Sequences 5′→3′	Final size (bp)	Annealing temp. (°C)
*Pvmdr*-*1* (OF)	CGCCATTATAGCCCTGAGCA	604	60
*Pvmdr*-*1* (OR)	TCTCACGTCGATGAGGGACT		
*Pvmdr*-*1* (NF)	GGATAGTCATGCCCCAGGATTG		57
*Pvmdr*-*1* (NR)	CATCAACTTCCCGGCGTAGC		
*Pvcrt*-*o* F	AAGAGCCGTCTAGCCATCC	296	52
*Pvcrt*-*o* R	AGTTTCCCTCTACACCCG		
*Pvcrt*-*o* (Rseq)	GGGGACGTCCTCTTGTATTT		55

### Evaluation of *pvcrt*-*o* K10 insertion by semi-nested PCR

Briefly in first round *pvcrt*-*o* (OF) and *pvcrt*-*o* (OR) primers (Table 1), were applied for amplification of about 1186 bp fragment of *pvcrt*-*o* gene of *P. vivax*. Three punched discs were used for each PCR reaction of the first round. One microlitre of all products of the first round diluted into 500 μl of sterile water. In the second round the 1 μl of *pvcrt*-*o*(OF) and *pvcrt*-*o* (Rseq) primers (Table 1) and 2 μl of diluted product of first round were added into 25 microlitre of “2X Taq Master Mix Red” (Amplicon Inc.), to reach a final concentration of 50 μl. The first round was done in 95 °C, 5 min; 30 cycles of 94 °C, 15 s; 52 °C, 30 s; 72 °C, 90 s; 72 °C, 7 min, while the second round PCR was done under the following conditions: 94 °C, 5 min; 30 cycles of 94 °C, 15 s; 55 °C, 30 s; 72 °C, 30 s; 72 °C, 7 min. The products of the second round (296 bp) were seen in 2% agarose gels (SYBR Safe stain; Invitrogen; Groningen, The Netherlands). The *Pvcrt*-*o*(OF) and PCR products were sent for sequencing by the ABI3730XL sequence analyzer (Macrogen, Korea) [8, 31].

### Development of asymmetric real time PCR and melt-curve analysis

#### Primer and probe design

**Fig. 2:**
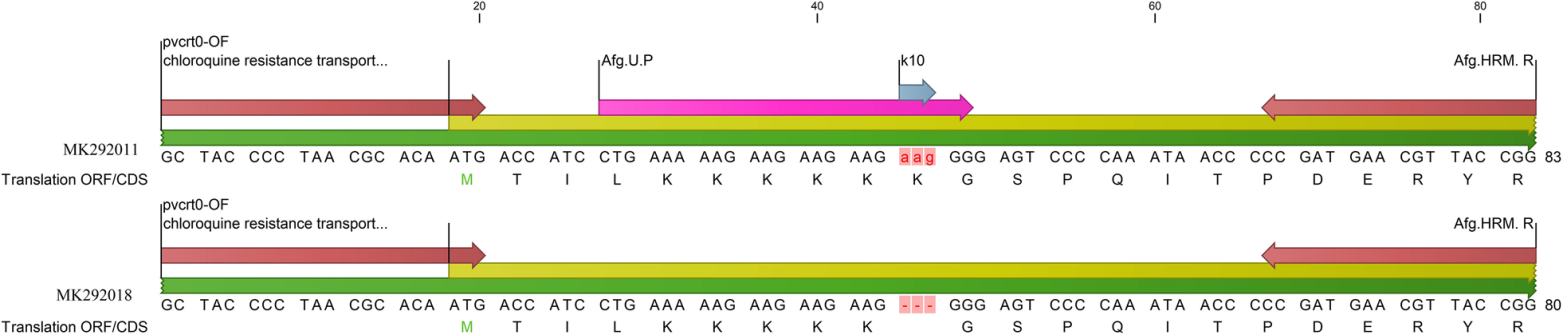
Schematic map of the *pvcrt*-o gene and applied primers and unlabelled probe in asymmetric qPCR: The position of primers (red flashes), unlabelled probe (purple arrow), K10 insertion (blue arrow), coding DNA sequence (yellow line) and mRNA (green line) illustrated by using of CLC bioinformatics software

The genomic sequence of *P vivax* (accession number: EU33972) was imported into CLC Main Workbench 5 (CLC bio, Aarhus, Denmark) Software. The *Pvcrto*-OF primer was selected as forward primer [8] and a reverse primer was designed for *pvcrt*-*o* gene. Then a probe designed that contain the insertion of interest. The probe blocking improved by amino-modified C6 to prevent extension during PCR amplification. The primers, probe sequences, and their position in the *pvcrt*-*o* gene are presented in Table 2 and Fig. 2.

**Table 2:** High-resolution melting assay primer and probe sequences used for detection K10 insertion in *pvcrt*-*o* gene

Name	Primer/probe	Sequence 5′→3′	TM	Products size	
				Wild	Mutant
Pvcrto-O F	Forward primer	GCTACCCCTAACGCACAATG	60	80	83
Afg.HRM. R	Reverse primer	CCGGTAACGTTCATCGG			
Afg.U.P	Unlabelled Probe	CTGAAAAAGAAGAAGAAGAAGGG- block

#### Amplification conditions

The qPCR-HRM assay was performed using an ABI 7500 Fast Real-time PCR system (Applied Biosystems, Inc.). The PCR was set up in a final volume of 20 µl containing 0.1 μM of Pvcrt0-OF (forward primer), 0.5 μM Afg.HRM. R (the reverse primer and the excess primer) and 0.5 μM Afg.U.P probe, 2 µl of diluted product of first round from semi nested PCR and 4 µl of 5X Hot Firepol^®^ EvaGreen^®^ HRM Mix (Solis BioDyne, Tartu, Estonia) in qPCR 8-strip tubes (Gunster Biotech, Taiwan).

The thermal program included an initial denaturation at 95 °C for 12 min, followed by 40 cycles of amplification consisting of 95 °C for 15 s (denaturation step), 60 °C for 20 s (primer annealing), and 72 °C for 20 s (elongation step). The amplicons were then subject to a melt program for dissociate the double stranded DNA at 95 °C for 15 s, gradual temperature for HRM increase from 55 °C for 1 min until 95 °C for 15 s at a thermal transition rate of 0.3%. Ultimately, obtained melting curve profiles were analysed by making use of HRM software for Windows^®^ version 3.0.1. (Applied Biosystems).

## Results

### Verification of *Plasmodium vivax* samples

All microscopically confirmed *P. vivax* isolated were also verified by semi-nested multiplex PCR and then applied for evaluation of mutations in *pvmdr1* and *pvcrt*-*o* genes.

### Evaluation *pvmdr1* mutations by nested-PCR

**Fig. 3:**
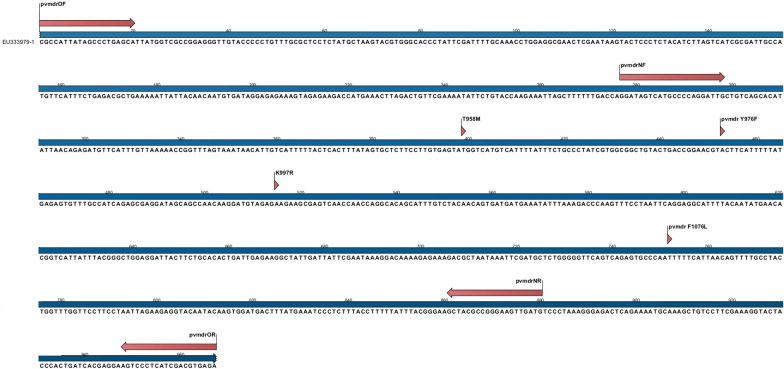
Schematic map of the *pvmdr*-*1* gene: The position of primers (red flashes) and mutations (red arrows) illustrated by using of CLC bioinformatics software

The *pvmdr*-*1* gene was successfully amplified and sequenced in 92% (33/36) of the *P. vivax* isolates. In spite of resequencing, 3 (8%) of the samples had not acceptable sequencing results. The analysis of sequencing data of the *pvmdr*-*1* gene (Fig. 3) showed wild type Y976 and K997 and mutant M958 and L1076 in all isolates from three provinces and submitted to GenBank, MK419882-MK419914 (Table 3).

**Table 3:** Distribution of *pvmdr1* mutations among *P. vivax* isolates from Laghman, Baghlan and Khost

Mutations (SNP)	No. isolates (%) from the following provinces			Frequency (%)
	Laghman	Baghlan	Khost	
T958M (ACG/ACG)	17 (100)	9 (100)	7 (100)	33 (100)
Y976F (TAC/TTC)	0 (0)	0 (0)	0 (0)	0 (0)
K997R (AAG/AGG)	0 (0)	0 (0)	0 (0)	0 (0)
F1076L (TTT/CTT)	17 (100)	9 (100)	7 (100)	33 (100)
Total	17	9	7	33 (100)

### Evaluation of K10 insertion in pvcrt-o gene by unlabelled probe and semi-nested PCR

The *pvcrt*-*o* gene was successfully amplified and sequenced in 100% (36/36) of the *P. vivax* isolates. Of the 36 samples evaluated for K10 insertion in *pvcrt*-*o*, 2/18(11%), 0/10(0%) and 0/8(0%) isolates from Laghman, Baghlan and Khost province possessed K10 insertion, respectively, that confirmed by either sequencing and unlabelled probes and submitted to GenBank, MK292011–MK292046 (Table 4).

**Table 4:** Distribution of *pvcrt*-*o* K10 insertion among *P. vivax* isolates from Laghman, Baghlan and Khost

Polymorphism	No. isolates (%) from the following provinces			Frequency (%)
	Laghman	Baghlan	Khost	
K10 Insert	2 (11.11)	0 (0)	0 (0)	2 (5.55)
total	18	10	8	36 (100)

### High-resolution melting assay primer and Unlabelled probe

**Fig. 4:**
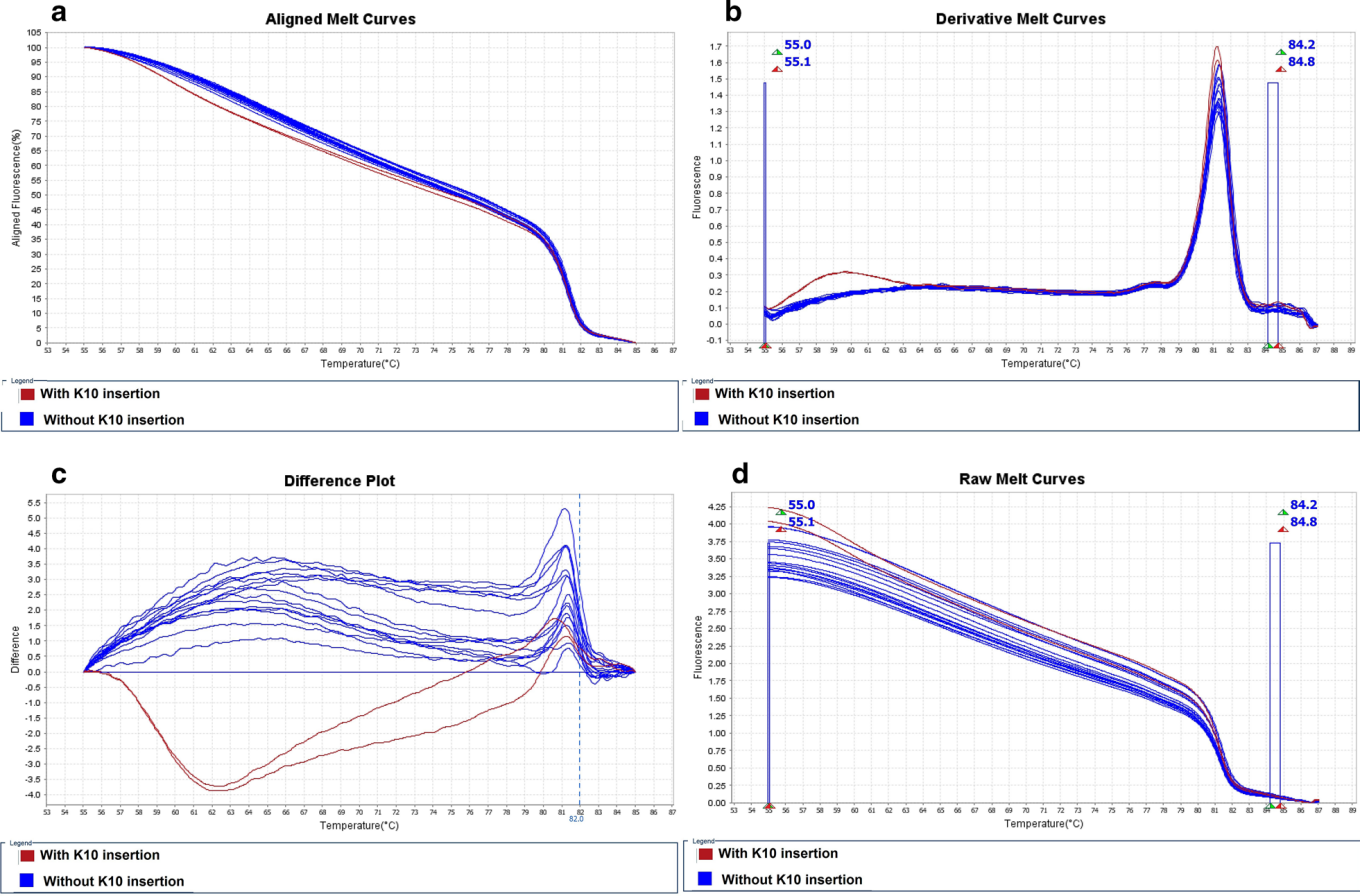
Comparison of melting temperature of *pvcrt*-*o* gene mutation by unlabelled probe: **a** Aligned and normalized melt curves, **b** derivative melt curves, **c** normalized difference plot and **d** Raw Melt curve respectively. Separation of inserted (red) and non-inserted (blue) K10 variation

Simultaneous amplification and screening of mutant and wild types of *pvcrt*-*o* gene was performed by qPCR-HRM and unlabelled probe. Unfortunately, PCR product melting transitions were not able to identify independently the genotype. The whole melting profile, showing the melting region of both the unlabelled probe and the PCR products, is shown in Fig. 1. Two peaks were apparent. If K10 insertion existed, the probe melted at 55.5–64 °C, whereas the PCR product melted at 78.5–83 °C (Fig. 4).

## Discussion

Malaria is endemic in the most parts of Afghanistan, and more than 95% of reported cases are *P. vivax*, for which chloroquine is used as first-line therapy [1]. Therefore, investigations on the frequency of mutations in the genes that are responsible for chloroquine resistance, such as *pvcrt*-*o* and *pvmdr1*, are needed. So far, evaluation of these mutations had not been carried out in Afghanistan and this work is the first report dealing with those mutations related to chloroquine resistance.

Y976F mutation in the *pvmdr1* gene as well as the K10 insertion in the *pvcrt*-*o* gene are considered responsible for *P. vivax* resistance to chloroquine [21, 23]. The Y976F mutation is present in high-level chloroquine-resistant foci (Thailand and Myanmar) compared with low-grade regions (Republic of Korea) [32]. In a research study done in Thailand, the Y976F and the F1076L mutations were reported 23% and 53% cases, respectively [33]. In the present study, as well as the investigations that were carried out in the Republic of Korea, Madagascar and Mauritania, the F1076L mutation was reported in all the isolates studied [22, 32, 34]. Although Y976F and K997R mutations were not observed in any of the *P. vivax* isolates from Afghanistan, the presence of two mutations F1076L and T958M in all isolates suggests a change in parasite genotype, which may lead to a modest positive change to reach the CQ resistant phenotype.

The insertion of AAG (codon of Lysine) was present in 56.0% and 46.2% of isolates from Thailand and Myanmar, respectively. The presence of K10 insertion in two isolates from Laghman province, which has shown an increasing trend in cases of *P. vivax* in the last 10 years, is alarming, which should lead the Ministry of Public Health of Afghanistan to investigate the efficacy of chloroquine in order to improve the treatment of malaria in the country.

In several studies conducted on the *pvcrt*-*o* gene, appropriate internal primers that can replicate K10 insertion were not used, and these primers replicated on the points further, such mutations did not exist at all [8, 22, 33]. In addition, due to the low copy number of this gene in *P. vivax*, the use of one pair of primers for amplification of the K10 insertion is not suitable for samples with low parasite loading [31].

In this study, a modified semi-nested PCR method was used to overcome those problems. In this method, two primers designed by Lu et al. were used for the first amplification of *pvcrt*-*o* gene [31]. The forward primer of first round and the *Rseq* primer of Glossa et al. were used in the second round [8]. Finally, sharp bands were seen suitable for sequencing. Although the sequencing is the most accurate method to detect SNPs, it is expensive, time consuming and in some circumstances impossible due to lack of infrastructures. The PCR–RFLP method is cheaper and faster, but lacks sufficient enzymes for all mutations. Melt curve analysis and the use of the unlabelled probe in asymmetric qPCR is a new method used in various studies. The use of this method is much affordable since unlabelled probes are cheaper than restriction enzymes and can be transferred at room temperature. Although, HRM analysis may be enough to detect mutations, the use of an unlabelled probe signifies the accuracy of the work. The qPCR does not have post-PCR manipulation problems, which are seen in DNA sequencing and PCR–RFLP. Due to financial problems, the investigation on all mutations by using the unlabelled probe method and examination of the K10 insertion was impossible.

Afghanistan is a country with other challenges, and it was not possible to investigate the copy number of the genes, to perform in vitro studies, or to study the clinical responses to *P. vivax* isolates. In fact, this work is a snapshot of CQ resistance of *P. vivax* situation in three provinces of Afghanistan. Further research needs to be performed on a larger number of samples throughout Afghanistan. There is a hope that the results of this study will be the basis for future investigations. To study the actual relationship between the mutations in the *pvmdr1* and *pvcrt*-*o* genes and drug resistance, the effect of these mutations in the corresponding proteins may be studied using molecular dynamics and protein modelling, with an analysis of the possible interactions with chloroquine in silico. Genomic, transcriptomic and proteomic studies would then give a better picture of these interactions.

## Conclusions

This study is an example of a study providing baseline data that may contribute to the discussion regarding the neglected status of chloroquine resistance of *P. vivax* in the region and subsequently on a global scale. The number of samples was relatively small and the results need to be taken with caution. Further in vitro and clinical observation should be done for a better understanding of chloroquine resistance in Afghanistan.

## Data Availability

All datasets are included within the paper. Any question may be obtained from the corresponding author upon request.

## Abbreviations

*Pvcrt*-*o*: *Plasmodium vivax* chloroquine resistant transporter
*Pvmdr*-*1*: *Plasmodium vivax* multidrug resistant
HRM: High Resolution Melting
qPCR: Quantitative Polymerase Chain Reaction
DBCs: DNA Banking Cards
RFLP: Restriction fragment length polymorphisms
